# Adherence to breast and cervical cancer screening guidelines according to obesity status: a French cross-sectional multicenter survey

**DOI:** 10.1016/j.pmedr.2026.103405

**Published:** 2026-02-09

**Authors:** Elise Foucault, Valérie Macioce, Marion Soler, Yves-Marie Pers, Jean-Baptiste Bonnet, Antoine Avignon, Nicolas Chevalier, Ariane Sultan

**Affiliations:** a Department of Nutrition and Diabetes, CHU Montpellier, Univ Montpellier, 371 Avenue du Doyen Gaston Giraud, 34295 Montpellier, France; b Clinical research and epidemiology unit, CHU Montpellier, Univ Montpellier, 39 avenue Charles Flahault, 34295 Montpellier, France; c IRMB, University of Montpellier, Inserm U1183, CHU Montpellier, Montpellier, France; d UMR 1302 Institute Desbrest of Epidemiology and Public Health, INSERM, Univ Montpellier, Montpellier, France; e Université Côte d'Azur, CHU, INSERM U1065, C3M, Nice, France; f Inserm U 1046, Physiology and Experimental Medicine, Heart and Muscles, 34295 Montpellier, France

**Keywords:** Early detection, Gynecological cancer, Diagnostic screening programs, Breast neoplasms, Uterine cervical neoplasms, Obesity, Body mass index

## Abstract

**Objective:**

To assess breast and cervical cancer screening rates among women according to body mass index (BMI).

**Methods:**

This cross-sectional study used anonymous self-administered questionnaires given to women from three French university hospitals with normal weight (BMI 18.5–25 kg/m^2^), class I obesity (BMI 30–35 kg/m^2^), or class II obesity (BMI ≥ 35 kg/m^2^) in 2020–2021. Up-to-date screening was defined according to national guidelines: biennial clinical breast exams and mammograms for women aged 50–74, and triennial cervical samples for women aged 25–65, excluding those with prior cancer or related surgery. Screening rates and gynecological follow-up were compared across BMI groups.

**Results:**

Among 439 women (20% class I, 22% class II obesity), 178 and 370 were eligible for breast and cervical cancer screening, respectively. Women with class I and II obesity were less likely to be up-to-date for clinical breast exams (Odds Ratio [95% confidence interval] 2.35[1.06,5.20] and 2.68[1.12,6.42], respectively), mammography (4.43[1.49,13.18] and 4.08[1.22,13.62]), and cervical samples (2.23[1.09,4.54] and 2.85[1.42,5.72]). Class II obesity was associated with more frequent follow-up by general practitioners (*p* < 0.05).

**Conclusion**s**:**

Women with obesity are less likely to receive recommended gynecological cancer screenings than normal-weight peers. These disparities call for more inclusive healthcare strategies.

**Trial registration**: NCT04357652.

## Highlights

• Both breast and cervical cancer screening were assessed across body mass index groups.
• Obesity (class I and II) was linked to breast and cervical cancer underscreening.
• Odds of non-up-to-date screening was two-to-four times higher in women with obesity.
• In class-II obesity, general practitioner more often led gynecological follow-up.
• Screening disparities were not explained by socioeconomic status.

## 1 Introduction

Obesity is a major public health issue, affecting 14% of adults worldwide (WHO, 2022). It is a risk factor for cardiovascular, renal, bone and joint diseases and diabetes, and is associated with certain cancers, particularly hormone dependent ones (Bhaskaran et al., 2014; GBD Obesity Collaborators et al., 2017). The Center of Disease control and prevention identified 13 obesity-related cancers, including breast and uterus (CDC, 2025). Excess weight contributes to postmenopausal breast cancer, with risk increasing by 1.1 times for every five additional units of body mass index (BMI) (Engmann et al., 2017; Lauby-Secretan et al., 2016). Among women with obesity, higher BMI is also associated with more invasive breast cancers and higher breast cancer mortality (Neuhouser et al., 2015). Obesity is also a risk factor for endometrium cancer with relative risk ranging from 2.5 to 7.1 depending on obesity class (Lauby-Secretan et al., 2016). Concerning cervical cancer, a few studies suggested a link between obesity and increased risk of cervical intraepithelial lesions (Okoro et al., 2020). A 2016 meta-analysis found a positive association between cervical cancer and obesity but highlighted divergent results between studies (Poorolajal and Jenabi, 2016).

In France, guidelines recommend breast cancer screening by mammography and clinical breast exam every two years for women aged 50–74, excluding high-risk women (HAS, 2015). European guidelines are similar, targeting women aged 50–69 for regular mammography, stating lower evidence for women aged 40–49 and 70–74 years (Cardoso et al., 2019). Conventional two-dimensional mammography remains the gold standard for screening, with sensitivity increasing with BMI (Njor et al., 2016). The relationship between obesity and screening guidelines adherence remains unclear. Some large-scale studies showed reduced mammography screening in women with obesity (Bussiere et al., 2014; Hellmann et al., 2015; Katz et al., 2018; McBride et al., 2024) while others did not (Ludman et al., 2010; Miles et al., 2019). A 2010 review already highlighted discordant results (Aldrich and Hackley, 2010).

For cervical cancer, French screening modalities for women aged 25–30 include two cytological examinations one year apart and then every three years if results are normal. From 30 to 65 years, a first-line Human Papillomavirus (HPV) test is advised three years after the last normal cytological examination and then every five years. This screening program aligns with European and World Health Organization guidelines, which propose HPV testing as the primary screening tool from age 35 to 60–65, while cytological examinations can be maintained for ages 20–30 (Chrysostomou et al., 2018).

Two reviews found obesity associated with a lower cervical cancer screening rate (Aldrich and Hackley, 2010; Maruthur et al., 2009). Most studies were carried out in Caucasian populations and some of them linked only severe obesity to reduced screening. Recent studies confirmed association between non-up-to-date Pap test screening and obesity (Bussiere et al., 2014; Ludman et al., 2010; Sassenou et al., 2021) or severe obesity (Perez et al., 2020). However, most focused on one cancer type.

Screening for these common cancers is a public health issue involving several professionals. Identifying who performs the screening may reveal women's care pathways and possible avoidance of certain providers. The main objective was to compare breast and cervical cancer screening rates in targeted age groups between women with class I/II obesity and normal weight. We hypothesized that screening rates would decrease with increasing BMI. Secondary objectives were to identify the professionals involved and compare results across BMI groups.

## 2 Methods

### 2.1 Study design and population

We conducted a cross-sectional multicenter study, reported according to STROBE guidelines (EQUATOR Network). The project was approved by the Montpellier University Hospital Institutional Review Board (#IRB-MPT_2020_03_202000398) and registered on clinicaltrials.gov (NCT04357652). Informed consent was not required under French regulation, but patients could decline participation.

All women aged 25–74 meeting inclusion criteria were screened at Montpellier, Nîmes, and Nice University Hospitals (Endocrinology/Nutrition, Bariatric Surgery, or Rheumatology departments) between October 2020 and April 2021. These departments were chosen to recruit patients with obesity without over-representation of cardiovascular or neoplastic complications. The COVID-19 pandemic temporarily slowed recruitment, but inclusions were not interrupted during the study. Women with obesity (BMI ≥30) were included: class I (30–34.9) and class II or above (≥35, summarized as Class II). Normal-weight women (BMI 18.5–25) served as controls, while overweight and underweight women were excluded. Additional inclusion criteria followed guidelines: for cervical cancer, women 25–65 without prior cancer or cervical surgery; for breast cancer, women 50–74 without prior cancer or breast surgery. Eligible women were informed during consultations and invited to complete an anonymous self-questionnaire.

### 2.2 Cancer screening assessment

Participants were assessed using an anonymous 19-item self-questionnaire (Appendix A), requiring about five minutes to complete. It covered socio-demographic data (age, sex, socio-professional group, health system aids), medical history, gynecological data (contraception, pregnancies, in vitro fertilization, menopause, surgery), and breast/cervical cancer screening. Socio-professional group followed categories of the National Institute of Statistics and Economic Studies (INSEE) or unemployed/retired/other. For screening, women aged 50–74 were asked about mammography or clinical breast exam in the past two years; a positive response was considered up-to-date per French guidelines (HAS, 2015). Women aged 25–65 were asked if they had a cervical sample (Pap smear or HPV test) in the past three years; a positive response was considered up-to-date per guidelines. They were also asked which professional managed their gynecological follow-up (general practitioner, gynecologist, endocrinologist, midwife, or other).

If breast or cervical cancer screening was not up to date according to current guidelines, participants were advised to undergo appropriate screening.

### 2.3 Statistical analysis

Analyses were carried out on two populations depending on the gynecological cancer considered. Populations were described with means and standard deviations (SD) for quantitative variables and numbers and percentages for qualitative variables. Continuous variables were tested for normality using the Shapiro-Wilk test, and compared between three BMI groups (normal weight, class I and class II obesity) with the Kruskal-Wallis test or ANOVA. Qualitative variables were compared using Chi^2^ or Fisher's exact test. Significant differences between groups were followed by pairwise comparisons with Benjamini-Hochberg correction; corrected *p*-values are reported.

Screening rates for each cancer were described, with bivariate analysis according to screening status (up-to-date or not). Multivariable logistic regression was performed, with age as a continuous variable. Odds ratios (OR) and 95% confidence intervals (CI) were calculated. Missing data were not replaced. Analyses were conducted by the clinical and epidemiological research unit of Montpellier University Hospital. Statistical significance was set with a p-value ≤0.05 using SAS Enterprise Guide 8.2 (SAS Institute, Cary, NC, USA).

## 3 Results

### 3.1 Whole study population

**Fig. 1:**
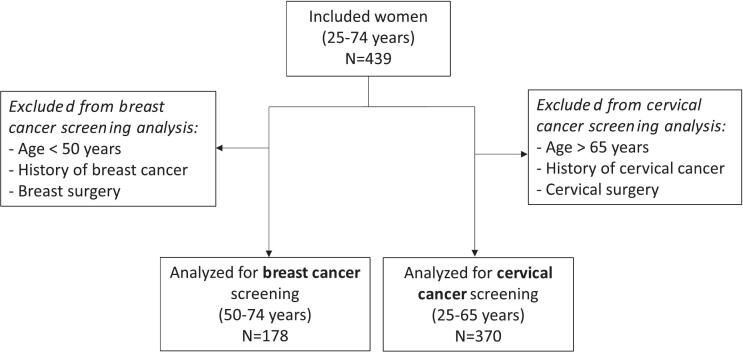
Flow chart of the study population (2020–2021 French online survey).

Among women invited to participate, more than 90% accepted. We included 439 women, including 256 (58%) with normal weight, 89 (20%) with class I obesity and 94 (22%) with class II obesity. Among them, 178 women (41%) were analyzed for breast cancer screening and 370 (84%) for cervical cancer screening (Fig. 1). Characteristics of both populations are described and compared between BMI classes in Table 1, Table 3.

**Table 1:** Socio-demographic and clinical characteristics by body mass index class among women (age 50–74) participating in a French online survey (2020−2021) and analyzed for breast cancer screening.

	Normal-weight women (N) 18 ≤ BMI ≤ 25 (*N* = 94)	Class I obesity 30 < BMI < 35 (*N* = 49)	Class II obesity BMI ≥ 35 (*N* = 35)	*p*^a^
BMI (kg/m^2^), *mean ± SD*	22.5 ± 2.0	31.9 ± 1.5	39.8 ± 4.1	< 0.01
Age (years), *mean ± SD*	60.6 ± 6.5	60.0 ± 6.5	58.6 ± 6.9	0.26
Socio-professional category				0.62
Higher intellectual professions and intermediary professions	24 (25.8)	8 (16.3)	5 (14.7)	
Employees, workers	25 (26.9)	13 (26.5)	10 (29.4)	
Unemployed, retired	40 (43.0)	23 (46.9)	16 (47.1)	
Shopkeepers, craftsmen, business leaders, others	4 (4.3)	5 (10.2)	3 (8.8)	
Heath system status/aid				
Long-term illness	36 (38.7)	27 (55.1)	18 (52.9)	0.12
Health system assistance b	4 (4.3)	3 (6.1)	5 (14.3)	0.13
Adult disability allowance	3 (3.2)	4 (8.3)	4 (11.8)	0.13
Medical history				
Diabetes	17 (18.3)	19 (38.8)	16 (45.7)	<0.01 ^N<I, II^
Cardiovascular disease	6 (6.5)	5 (10.4)	6 (17.6)	0.17
Osteoarthritis	15 (48.4)	9 (64.3)	12 (85.7)	0.06
Cervical cancer	2 (2.2)	2 (4.1)	1 (2.9)	0.84
Polycystic ovary syndrome	5 (5.4)	1 (2.0)	1 (2.9)	0.87
Number of pregnancies, *mean ± SD*	2.3 ± 1.5	2.4 ± 1.8	2.7 ± 1.8	0.52
Number of live births*, mean ± SD*	1.7 ± 1.1	1.9 ± 1.4	2.2 ± 1.7	0.45
Menopause	87 (93.5)	41 (85.4)	30 (85.7)	0.18
Menopause with hormone replacement therapy	11 (12.8)	5 (12.5)	0	0.09
Gynecological follow-up	87 (95.6)	45 (95.7)	28 (84.8)	0.09
Gynecological follow-up by				
General Practitioner	17 (18.7)	10 (21.3)	13 (39.4)	0.05 ^II>N, I^
Gynecologist	73 (80.2)	39 (83.0)	16 (48.5)	< 0.01
Endocrinologist	2 (2.2)	1 (2.1)	0	1.00
Midwife	2 (2.2)	0 (0.0)	1 (3.0)	0.59

Values are numbers (percentages) unless otherwise stated.

Abbreviations: BMI: body mass index; SD: standard deviation.

a p-value for Kruskall-Wallis, Chi 2 or Fisher exact test. The superscript letter (N) and figures (I, II) indicate which groups differ from each other (pairwise comparisons): N (normal weight women); I (class I obesity); II (class II obesity).

b Health system assistance: Universal Health Protection, Complementary Health Care, State Medical Aid.

### 3.2 Breast cancer screening population

#### 3.2.1 Socio-demographic and clinical data

Among women analyzed for breast cancer screening, main socio-demographic characteristics were similar across BMI groups (Table 1). History of diabetes was more frequent among women with class I and II obesity than in normal weight women (*p* = 0.01 and < 0.01, respectively). Women with class II obesity had their gynecological follow-up more often made by general practitioners and less often made by a gynecologist than women with normal weight (*p* < 0.01) and class I obesity (*p* < 0.01).

#### 3.2.2 Clinical breast exam

Women with normal weight tended to have more up-to-date clinical breast exam (68/88, 77.3%) than women with class I (27/45, 60%, *p* = 0.06) and class II obesity (20/34, 58.8%, *p* = 0.06).

After adjusting for potential risk factors (age, socio-professional category, health system assistance), women with obesity were significantly more at risk of not having up-to-date clinical breast exam than women with normal weight, with OR = 2.35 [95%CI: 1.06,5.20] for women with class I obesity and OR = 2.68 [1.12,6.42] for women with class II obesity (Table 2). No other factor was associated with this risk.

**Table 2:** Factors associated with breast cancer screening (clinical breast exam and mammography) among women (age 50–74) participating in a French online survey (2020–2021): multivariable analyses.

Variables	Unit or Modalities	Clinical breast exam not up to date (*n* = 52)	Clinical breast exam up to date (*n* = 115)	Multivariable model
				OR [95% CI]
Age (years)	Mean ± SD	59.7 ± 6.4	60.1 ± 6.5	0.99 [0.92,1.05]
BMI, n (%)	< 30	20 (38.5)	68 (59.1)	1
	[30–35[	18 (34.6)	27 (23.5)	2.35 [1.06,5.20]
	≥ 35	14 (26.9)	20 (17.4)	2.68 [1.12,6.42]
Socio-professional category, n (%)	Higher intellectual/intermediary professions	10 (19.2)	24 (21.1)	1
	Employees, workers	16 (30.8)	31 (27.2)	1.11 [0.41,2.99]
	Unemployed, retired	23 (44.2)	50 (43.9)	1.14 [0.42,3.12]
	Shopkeepers, others	3 (5.8)	9 (7.9)	0.67 [0.14,3.18]
Health system assistance, n (%)	No	50 (96)	105 (91)	1
	Yes	2 (3.8)	10 (8.7)	0.38 [0.07,1.93]
Variables	Unit or Modalities	Mammography not up to date (*n* = 24)	Mammography up to date (*n* = 150)	Multivariable model
				OR [95% CI]
Age (years)	Mean ± SD	60.3 ± 6.5	60.1 ± 6.6	0.99 [0.92,1.08]
BMI, n (%)	< 30	6 (25.0)	84 (56.0)	1
	[30–35[	11 (45.8)	38 (25.3)	4.43 [1.49,13.18]
	≥ 35	7 (29.2)	28 (18.7)	4.08 [1.22,13.62]
Socio-professional category, n (%)	Higher intellectual/intermediary professions	6 (25.0)	29 (19.5)	1
	Employees, workers	5 (20.8)	43 (28.9)	0.48 [0.13,1.82]
	Unemployed, retired	12 (50.0)	66 (44.3)	0.76 [0.22,2.60]
	Shopkeepers, others	1 (4.2)	11 (7.4)	0.30 [0.03,3.00]
Health system assistance^a^, n (%)	No	23 (95.8)	139 (92.7)	1
	Yes	1 (4.2)	11 (7.3)	0.54 [0.06,4.68]

Abbreviations: BMI: body mass index; CI: confidence interval; OR: odds ratio; SD: standard deviation. ^**a**^ Health system assistance: Universal Health Protection, Complementary Health Care, State Medical Aid.

#### 3.2.3 Mammography

Breast cancer screening by mammography was less frequently up to date in women with class I obesity (38/49, 77.6%) than in women with normal weight (84/90, 93.3%, *p* = 0.02). Mammography was up to date in 28/35 (80%) women with class II obesity, which was not different from the two other groups.

After adjusting for potential risk factors (age, socio-professional category, health system assistance), women with obesity were significantly more at risk of not having up-to-date mammography than women with normal weight, with OR = 4.43 [95%CI: 1.49,13.18] for women with class I obesity and OR = 4.08 [1.22,13.62] for women with class II obesity (Table 2). No other factor was associated with this risk.

### 3.3 Cervical cancer screening population

#### 3.3.1 Socio-demographic data

Among women analyzed for cervical cancer screening, women with class I obesity were older than women with normal weight (*p* = 0.02) (Table 3). Women with class II obesity included less “higher intellectual and intermediary professions” (*p* < 0.01) and more inactive or retired individuals (*p* = 0.04) than those with normal weight. As expected, women with class I and II obesity tended to benefit more often from the “long-term illness” health insurance status (*p* = 0.05 and 0.08) and needed more health system assistance (*p* = 0.01 and < 0.01) and adult disability allowance (*p* = 0.07 and 0.07) (Table 3).

**Table 3:** Socio-demographic and clinical characteristics by body mass index class among women (age 25–65) participating in a French online survey (2020–2021) and analyzed for cervical cancer screening.

	Normal-weight women (N) 18 ≤ BMI ≤ 25 (*N* = 217)	Class I obesity 30 < BMI < 35 (*N* = 73)	Class II obesity BMI ≥ 35 (*N* = 80)	*p*^a^
BMI (kg/m^2^), *mean ± SD*	22.1 (±2.0)	31.8 (±1.6)	41.1 (±5.7)	< 0.01
Age (years), *mean ± SD*	41.6 (±13.4)	46.3 (±11.3)	43.9 (±10.6)	0.01 ^N<I^
Socio-professional category				< 0.01
Higher intellectual professions and intermediary professions	71 (33.2)	14 (19.2)	10 (12.8)	N > II
Employees, workers	90 (42.1)	30 (41.1)	38 (48.7)	
Unemployed, retired	37 (17.3)	20 (27.4)	24 (30.8)	N < II
Shopkeepers, craftsmen, business leaders, others	16 (7.5)	9 (12.3)	6 (7.7)	
Heath system status/aid				
Long-term illness	62 (28.7)	32 (43.8)	31 (40.8)	0.03
Health system assistance b	10 (4.6)	10 (13.7)	16 (20.0)	< 0.01 ^N<I, II^
Adult disability allowance	6 (2.8)	7 (9.7)	7 (9.2)	0.01
Medical history				
Diabetes	34 (15.7)	18 (24.7)	29 (36.3)	< 0.01^N<II^
Cardiovascular disease	3 (1.4)	4 (5.6)	5 (6.6)	0.03
Osteoarthritis	11 (30.6)	6 (40.0)	12 (66.7)	0.04 ^N<II^
Breast cancer	9 (4.2)	0	2 (2.5)	0.22
Polycystic ovary syndrome	15 (6.9)	5 (6.8)	7 (8.8)	0.86
Number of pregnancies, *mean ± SD*	1.6 ± 1.6	2.3 ± 2.0	2.5 ± 2.0	< 0.01 ^N<I, II^
Number of live births*, mean ± SD*	1.1 ± 1.1	1.7 ± 1.4	1.8 ± 1.5	< 0.01 ^N<I, II^
Menopause	70 (32.6)	28 (38.4)	26 (32.5)	0.64
Menopause with hormone replacement therapy	10 (13.9)	3 (11.1)	1 (3.8)	0.50
Gynecological follow-up	211 (99.1)	69 (95.8)	74 (94.9)	0.05
Gynecological follow-up by				
General Practitioner	30 (14.1)	8 (11.1)	22 (28.2)	< 0.01 ^II>N, I^
Gynecologist	169 (79.3)	62 (86.1)	56 (71.8)	0.10
Endocrinologist	11 (5.2)	7 (9.7)	2 (2.6)	0.17
Midwife	30 (14.1)	5 (6.9)	10 (12.8)	0.28

Values are numbers (percentages) unless otherwise stated.

Abbreviations: BMI: body mass index; SD: standard deviation.

a p-value for Kruskall-Wallis, Chi 2 or Fisher exact test. The superscript letter (N) and figures (I, II) indicate which groups differ from each other (pairwise comparisons): N (normal weight women); I (class I obesity); II (class II obesity).

b Health system assistance: Universal Health Protection, Complementary Health Care, State Medical Aid.

#### 3.3.2 Medical history and gynecological data

Among women analyzed for cervical cancer screening, women with class II obesity had a more frequent history of diabetes (*p* < 0.01) and osteoarthritis (*p* = 0.03) than women with normal weight (Table 3). Women with class I and class II obesity had significantly more pregnancies (both *p* < 0.01) and more live births (both *p* < 0.01). No difference across BMI groups was observed in the proportion of women with menopause. Women with class II obesity had their gynecological follow-up more often made by general practitioners as compared with women with normal weight and class I obesity, whereas follow-up by a gynecologist did not differ significantly between BMI groups.

#### 3.3.3 Cervical cancer screening

Cervical cancer screening (Pap test or HPV test) was significantly more frequently up-to-date in women with normal weight (186/212, 87.7%) than in women with class I (52/69, 75.3%, *p* = 0.02) and II obesity (55/77, 71.4%, *p* < 0.01).

After adjustment for potential confounding factors (age, socio-professional category, health system assistance), the risk of non-up-to-date Pap test or HPV test was higher among women with class I and II obesity than among women with normal weight, with OR = 2.23 [95%CI 1.09,4.54 and 2.85 [1.42,5.72] (Table 4). No other factor was associated with this risk.

**Table 4:** Factors associated with cervical cancer screening (cervical sample for Pap test or Human Papillomavirus test) among women (age 25–65) participating in a French online survey (2020–2021): multivariable analysis.

Variables	Unit or Modalities	Cervical sample not up to date (*n* = 65)	Cervical sample up to date (*n* = 293)	Multivariable model
				OR [95% CI]
Age (years), mean ± SD		43.1 ± 12.7	42.8 ± 12.5	1.00 [0.98,1.02]
BMI, n (%)	< 30	26 (40.0)	186 (63.5)	1
	[30–35[	17 (26.2)	52 (17.7)	2.23 [1.09,4.54]
	≥ 35	22 (33.8)	55 (18.8)	2.85 [1.42,5.72]
Socio-professional category, n (%)	Higher intellectual/intermediary professions	16 (25.0)	77 (26.5)	1
	Employees, workers	24 (37.5)	133 (45.7)	0.68 [0.33,1.40]
	Unemployed, retired	14 (21.9)	61 (21.0)	0.75 [0.32,1.77]
	Shopkeepers, others	10 (15.6)	20 (6.9)	1.83 [0.68,4.91]
Health system assistance, n (%)	No	54 (83.1)	270 (9.2)	1
	Yes	11 (16.9)	23 (7.8)	1.46 [0.59,3.57]

Abbreviations: BMI: body mass index; CI: confidence interval; OR: odds ratio; SD: standard deviation.

## 4 Discussion

This multicenter study is original in analyzing screening status for two gynecological cancers, as well as the professionals involved. It shows that gynecological follow-up in women with obesity is insufficient and significantly less optimal than in normal-weight women, particularly for breast and cervical cancer screening. Women with class I and II obesity had four-fold higher odds of not being up-to-date for mammography and more than two-fold for clinical breast exam and cervical sampling. Unlike most studies focusing on a single cancer, we assessed both screenings, suggesting underscreening is linked to obesity itself rather than a specific cancer. These findings are consistent with data from the French CONSTANCES cohort (Franck et al., 2020; Franck et al., 2021). Our findings are striking since, unlike previous studies, BMI was associated with lower follow-up adherence, but socioeconomic status was not, despite its correlation with BMI (Sassenou et al., 2021).

We found differences in the professionals involved depending on women's weight status. In class II obesity, gynecological cancer screening was more often performed by general practitioners, while gynecologists were less often consulted. This underlines the need for education on weight bias to help improve screening.

Our results are consistent with those of a large French study reporting that probabilities of Pap test and mammography screening decreased by 24% and 23% in women with obesity compared to non-obese women (Bussiere et al., 2014). Nevertheless, we could have expected an improvement in the screening status of women affected with obesity since this study published in 2014, as several screening campaigns have been organized meanwhile. Previous studies also described a significant association between sociodemographic factors including education level and screening (Bussiere et al., 2014; von Euler-Chelpin et al., 2024), whereas in our study the socio-professional category was not associated with screening. Other studies have reported a significant association between increased BMI and decreased adherence to mammography screening (Aldrich and Hackley, 2010; Katz et al., 2018) and have suggested the importance of some health-professionals-related factors in patient adherence to screening, contrary to what we observed in our study.

However, contradictory results have been observed in the literature. In 2010 a literature review reported that only eight out of 15 studies observed under-screening by mammography in women with obesity, most of these studies being from the United States (Aldrich and Hackley, 2010). Our results mainly concerned Caucasian women with severe obesity. Some authors suggested that African-American women, with higher obesity prevalence, may have a less strict ideal of corpulence and therefore greater mammography use. Accordingly, seven studies found no screening difference by obesity status. Maruthur et al. also supported this for cervical cancer, as four studies in African-American women showed no significant association between obesity and under-screening (Maruthur et al., 2009). A more recent study from the United States also showed no association between obesity and under-screening by mammography (Miles et al., 2019). This may be due to improved care for women with obesity in the United States, where obesity affects nearly 40% of the population.

Our data on cervical cancer screening are also consistent with previously published studies. In 2009 a meta-analysis from the United States already showed a gradual under-screening for cervical cancer with increasing BMI (OR [95% CI] was 0.81 [0.70,0.93], 0.75 [0.64,0.88] and 0.62 [0.55,0.69] for women with obesity of grade I, II and III, respectively, compared to normal BMI) (Maruthur et al., 2009). In a literature review, Aldrich et al. found 13 of 17 studies concluding to significant under-screening for cervical cancer in women with obesity, mainly Caucasian women (Aldrich and Hackley, 2010). Indeed, as cervical cancer is a non-hormone dependent cancer, its potential association with obesity may not be due to pathophysiological pathways, but rather to a lower screening rate.

Under-screening for cervical cancer implies less detection of pre-cancerous lesions in women with obesity and therefore a later discovery of cervical cancer (Clarke et al., 2018). Recent changes in HPV screening modalities with the possibility of self-sampling could facilitate the identification of these lesions in women with obesity and their adherence to screening (HAS, 2019).

Previous studies (Aldrich and Hackley, 2010; Graham et al., 2022; Maruthur et al., 2009) raised hypotheses about the barriers to screening in women with obesity: negative body image, discrimination feeling associated with prejudices, management of comorbidities preceding screening, physical difficulties of gynecological examination and also lower quality of cervical sampling (e.g. vaginosis leading to non-interpretable results).

On the other hand, the impact of some healthcare providers that hold negative behavior and attitudes toward patients with obesity who present in healthcare settings must be emphasized (Graham et al., 2022).

Furthermore, the underscreening of gynecological cancers is also observed among people with diabetes (Bhatia et al., 2020) or in low socio economic status patients (Chan et al., 2014) diabetes, suggesting screening access inequalities.

General practitioners were more often responsible for gynecological follow-up in women with class I or II obesity than in normal-weight women. This may reflect easier access or stronger trust in family doctors, as noted in several studies (Aldrich and Hackley, 2010; Katz et al., 2018; Maruthur et al., 2009). Access to screening for women with obesity could be improved by strengthening relationships with healthcare providers. As they seem more confident with their general practitioner for gynecological follow-up, this primary-care provider may foster better adherence to screening guidelines than mobile programs delivered by unfamiliar providers (Atkins et al., 2013).

Several limitations should be noted. First, BMI, although routinely used, does not fully reflect obesity as it ignores body composition, and self-reported weight and height may bias BMI estimates. Self-reported screening could also cause recall bias, although this was limited by closed questions focused on the most recent screening. In addition, free access to screening for all women in France, compared with other countries, may reduce socio-economic bias. Another limitation is the lack of information on vaccination status. In France, HPV vaccination has been recommended since 2007, initially for girls and later extended to boys, targeting ages 11–14 with catch-up up to 19 years (French Health Ministry, 2021). At that time, women from our sample were aged 12–52, therefore a few women may be vaccinated. In France, screening guidelines do not differ according to HPV vaccination status. Moreover, we did not collect family history of cancer, which may affect risk and screening protocols. The impact of hormone replacement therapy could not be assessed due to the small number of women concerned. Finally, asking about screening within 3 years may have introduced bias, as some guidelines allow a 5-year interval for women aged 30–65.

In 2019, screening rates in the general French population were around 60% for cervical cancer and 48.6% for breast cancer (HAS, 2019; Santé publique France, 2020). In our study, rates were higher: 81.8% for cervical cancer; 86.2% for mammography and 68.8% for clinical breast examination. This may reflect recruitment in healthcare settings, involving women already under medical supervision, or recall bias about recent screenings. As all BMI groups came from the same population, this should not have affected results. Still, the improvement in screening rates is greater in women with normal BMI than in those with obesity. Finally, the Covid-19 pandemic beginning in March 2020 likely delayed care and screening, potentially increasing under-screening, particularly among women with obesity who were more vulnerable during this period.

In conclusion, women with obesity experience substantial under-screening for breast and cervical cancers. Addressing weight stigma among health professionals and strengthening the role of general practitioners could improve screening uptake, while HPV self-sampling may facilitate access for women less comfortable with gynecological examinations.

## CRediT authorship contribution statement

**Elise Foucault:** Writing – original draft, Investigation, Conceptualization. **Valérie Macioce:** Writing – review & editing, Writing – original draft, Methodology. **Marion Soler:** Writing – review & editing, Formal analysis, Data curation. **Yves-Marie Pers:** Investigation. **Jean-Baptiste Bonnet:** Writing – review & editing, Investigation. **Antoine Avignon:** Investigation. **Nicolas Chevalier:** Writing – review & editing, Investigation. **Ariane Sultan:** Writing – review & editing, Writing – original draft, Supervision, Investigation, Conceptualization.

## Funding

This research did not receive any specific grant from funding agencies in the public, commercial, or not-for-profit sectors.

## Declaration of competing interest

The authors declare that they have no known competing financial interests or personal relationships that could have appeared to influence the work reported in this paper.

## Data Availability

Data will be made available on request.

## Appendix A Supplementary data

Supplementary material Image 1
