# Supplementary material for: Adherence to breast and cervical cancer screening guidelines according to obesity status: a French cross-sectional multicenter survey
Source: Prev Med Rep. 2026 Feb 9;63:103405. doi: 10.1016/j.pmedr.2026.103405 (PMC12925188; doi:10.1016/j.pmedr.2026.103405)
Supplement: Supplementary file 1 — Supplementary material [file mmc1.docx]

**Appendix A: anonymous self-questionnaire given to participating women (2020-2021 French online survey)**

*Translation of the patient questionnaire from French to English.*

Dear Madam,

As part of a thesis coordinated by Pr SULTAN at Montpellier University Hospital, we would like to compare access to gynaecological care (screening for gynaecological cancers and prescription of contraception) between women with obesity and women with normal weight.

Thank you for taking the time to complete this anonymous questionnaire.

You are aged between 25 and 74:

1. Your age : ………… Your weight: …………. Your height : …………...
2. Which socio-professional category do you belong to?

- Farmers
- Shopkeepers, craftsmen, business leaders
- Higher intellectual professions
- Intermediary professions
- Workers
- Employees
- Unemployed, retired
- Ohers : …………………………………………………………………………

1. Do you benefit from the following social or health system assistance (one or several possible answers) :

- Long-Term Illness
- Universal Health Protection
- Complementary Health Care
- State Medical Aid
- Adult Disability Allowance
- Earned Income Supplement

1. Do you have any history of : (several possible answers)

- diabetes
- cardiovascular disease
- rheumatismal disease
- inflammatory rheumatism
- osteoarthritis
- renal disease
- psychiatric disease
- breast cancer
- cervical cancer
- polycystic ovary syndrome

1. Have you ever had obesity surgery

- yes
- no

if yes, when (year) ? ……………………..

1. Have you ever had gynaecological surgery (hysterectomy, tubal ligation,…) ?

- yes
- no

If yes, which kind of surgery ? …………………………………………………………………….

1. How many pregnancies have you had? …………...

Among these pregnancies, can you tell us how many were:

- Alive birth : ……
- Termination of pregnancy : ……
- Miscarriage : …….

Have you ever had non-desired or non-planed pregnancies ?

- yes
- no

Have you already undergone *In Vitro* Fertilisation ?

- yes
- no

1. Are you menopausal ?

- yes
- no

1. If yes, have you been or are you on hormone replacement therapy?

- yes
- no

Who prescribed it ?

- general practitioner
- gynecologist
- endocrinologist
- other : ……………………………………………………

1. Who is in charge of your gynaecological follow-up? (one or several possible answers)

- general practitioner
- gynecologist
- endocrinologist
- midwife
- other : ……………………………………………………

1. This (these) practitioner(s) is (are) :

- A female
- A male
- Female and male

1. As part of your gynaecological follow-up, your general practitioner :

- has already **given you information** about pap smear tests and mammography (e.g. how often and how they are carried out) ?

- yes
- no

- has already **carried out your** pap smear screening test?

- yes
- no

- has already **carried out your gynaecologic or clinical breast exam** ?

- yes
- no

- has already **referred you** to other specialists for your gynaecological follow-up?

- yes
- no

- has already **prescribed you contraception**?

- yes
- no

- has already **placed** a contraception ?

- yes
- no

- has already **spoken** to you about or **prescribed hormone replacement therapy** for the menopause?

- yes
- no

1. If you are aged between 25 and 65, have you had a cervical pap smear or HPV (Human Papillomavirus) test in the last three years?

- yes
- no

Who carried out this test ?

- general practitioner
- gynecologist
- endocrinologist
- midwife
- other : ……………………………………………………

1. If you are aged between 50 and 74, have you had a mammography in the last two years?

- yes
- no

who prescribed it to you ?

- general practitioner
- gynecologist
- endocrinologist
- no prescription because invitation for screening received
- other : ………………………………………………………………

1. If you are aged between 50 and 74, have you had a clinical breast exam in the last two years?

- yes
- no

Who carried it out ?

- general practitioner
- gynecologist
- endocrinologist
- midwife
- other : ……………………………………………………

1. If you are not menauposal :

Do you need a contraception ?

- yes
- no

Do you use a contraception ?

- yes
- no

🡪 if yes, can you specify the type and, if possible, the name of the contraception?

- Contraceptive pill : …………………………………………………
- Intra uterine device (IUD) (if possible specify hormonal or copper IUD in addition to the IUD name) : ………………………………………
- Implant : ……………………………………………………………
- Patch : ……………………………………………………………...
- Vaginal ring : ………………………………………………….
- Male condom
- Female condom
- Diaphragm

Who prescirbed it to you ?

- general practitioner
- gynecologist
- endocrinologist
- midwife
- other : ……………………………………………………

If you have an IUD, implant, etc, who placed it ?

- general practitioner
- gynecologist
- endocrinologist
- midwife
- other : ……………………………………………………

🡪 If no, are you planning a pregnancy ?

- yes
- no

1. On a scale ranging from 0 to 10, how do you rate your feelings at the time of a gynaecological examination? (0 = no discomfort and 10 = unbearable discomfort)

0 1 2 3 4 5 6 7 8 9 10

1. When you discuss about gynaecological follow-up with your doctor and during the clinical examination, you find him/her :

- Very comfortable
- Comfortable
- Uncomfortable
- I do not want to answer
- I have no opinion on this subjects

1. Concerning your gynecological follow-up, do you feel a difference in care linked to your weight? (0 = no difference felt and 10 = major difference felt)

0 1 2 3 4 5 6 7 8 9 10

Madam, thank you for answering this questionnaire.
